# Amyloid-beta peptide and phosphorylated tau in the frontopolar cerebral cortex and in the cerebellum of toothed whales: aging versus hypoxia

**DOI:** 10.1242/bio.054734

**Published:** 2020-11-05

**Authors:** Simona Sacchini, Josué Díaz-Delgado, Antonio Espinosa de los Monteros, Yania Paz, Yara Bernaldo de Quirós, Eva Sierra, Manuel Arbelo, Pedro Herráez, Antonio Fernández

**Affiliations:** 1 Veterinary Histology and Pathology, Institute of Animal Health, University of Las Palmas de Gran Canaria, Veterinary School, c/Transmontaña s/n, 35416 Arucas; 2 Laboratory of Wildlife Comparative Pathology (LAPCOM), School of Veterinary Medicine and Animal Science, University of São Paulo, São Paulo, 05508-270 SP, Brazil; 3 Texas A&M Veterinary Medical Diagnostic Laboratory, Pathology Division, College Station, TX 77843, USA

**Keywords:** Toothed whales, Beaked whales, Hypoxia, Beta amyloid, Phosphorylated tau

## Abstract

Hypoxia could be a possible risk factor for neurodegenerative alterations in cetaceans’ brain. Among toothed whales, the beaked whales are particularly cryptic and routinely dive deeper than 1000 m for about 1 h in order to hunt squids and fishes. Samples of frontal cerebral and cerebellar cortex were collected from nine animals, representing six different species of the suborder Odontoceti. Immunohistochemical analysis employed anti-β-amyloid (Aβ) and anti-neurofibrillary tangle (NFT) antibodies. Six of nine (67%) animals showed positive immunolabeling for Aβ and/or NFT. The most striking findings were intranuclear Aβ immunopositivity in cerebral cortical neurons and NFT immunopositivity in cerebellar Purkinje neurons with granulovacuolar degeneration. Aβ plaques were also observed in one elderly animal. Herein, we present immunohistopathological findings classic of Alzheimer's and other neurodegenerative diseases in humans. Our findings could be linked to hypoxic phenomena, as they were more extensive in beaked whales. Despite their adaptations, cetaceans could be vulnerable to sustained and repetitive brain hypoxia.

## Abstract (precis)

**Summary:** Immunohistochemical analysis showed positive immunolabeling for amyloid-beta peptide and phosphorylated tau in the cerebral and cerebellar cortex of toothed whales. These findings could be linked to hypoxic phenomena.

## INTRODUCTION

Over 46 million people live with dementia worldwide and this number is estimated to attain global epidemic dimensions (131.5 million people) by 2050 (Prince et al., 2015). Alzheimer's disease (AD) is a complex, multifactorial disease for which a number of genetic, environmental, and lifestyle risk factors have been identified (Iqbal and Grundke-Iqbal, 2010). Its pathologic hallmarks are represented by the accumulation of beta-amyloid (Aβ) and neurofibrillary tangles (NFT) in neurons, neuroglia and neuroparenchyma, leading to neuronal and synaptic loss, and eventual brain atrophy (Sun et al., 2015). Vascular contributions to cognitive impairment and dementia have also been linked to AD (Snyder et al., 2015). Decreased blood flow to the central nervous system (CNS) in humans is classically associated with AD-related pathology and alterations in the CNS vasculature could impair clearance of Aβ, and thereby accelerate the progression of AD (Koike et al., 2010).

NFT are composed of insoluble paired helical filaments of a highly phosphorylated form of the microtubule-associated protein τ (tau) and associated lipid. While typical NFT have been reported in senior cats, increased phosphorylation of τ without typical NFT has been described in sheep and goat (*Cetartiodactyla*), cat, dogs, leopards, cheetah, bison, degu, wolverine, bear and American bison (Youssef et al., 2016). In these cases, the τ changes tend to be sporadic and do not adopt ‘full blown’ features as seen in humans (Youssef et al., 2016). Again, evidence that Aβ pathology increases with age has been reported in a large number of non-human primate species: New and Old World monkeys, (Geula et al., 2002; Lemere et al., 2008; Youssef et al., 2016) and some great apes like the western lowland gorilla (Kimura et al., 2001; Perez et al., 2013). On the contrary, NFT have rarely been described in non-human primate brain with the exception of a single aged chimpanzee (Rosen et al., 2008) and in the gray mouse lemur (Bons et al., 2006). Interestingly, some potential longevity in humans is much longer than great apes (40 to 55 years) (Perez et al., 2013) or even some species like the gray mouse lemur (*Microcebus murinus*), which is considered to be elderly over 5 years of age (Bons et al., 2006).

Natural animal models of AD should recapitulate two major histopathological hallmarks: Aβ deposition and NFT formation. However, there are no natural animal models with comparable neurodegenerative findings. The choice of an ideal animal natural model is hampered by which animals appear to show species-specific variations in the process of phosphorylation and cleavage of tau protein (Sarasa and Pesini, 2009). Cetaceans are homeotherms and long-lived top predators, which are at high risk of bioaccumulation and biomagnification of a variety of chemical pollutants (Stavros et al., 2007). The maximum-recorded lifespan in the killer whale (*Orcinus orca*) is 78 years, in the short-finned pilot whale it is 63 years, and in the striped dolphin it is 58 years (Foster et al., 2012; Gunn-Moore et al., 2017). Recent studies have drawn some novel attention to neurodegenerative diseases (NDD) in cetaceans and it has been suggested that dolphins might be one of the very few potential natural models of AD (Davis et al., 2019; Di Guardo, 2018a,b, 2019; Gunn-Moore et al., 2017; Sacchini et al., 2018; Sarasa and Pesini, 2009; Stylianaki et al., 2019). Positive labelling to τ was observed in the cytoplasm of neurons of deep cortical areas of frontal, parietal and temporal lobe of one bottlenose dolphin (*Tursiops truncatus*) with no clear NFT (Stylianaki et al., 2019). On the other hand, well-defined NFT were observed in the frontal and parietal cortex, thalamus and cerebellum of one bottlenose dolphin and three striped dolphins (*Stenella coeruleoalba*) (Gunn-Moore et al., 2017). Aβ plaques were also observed in the striped dolphin, bottlenose dolphin, and common dolphin (*Delphinus delphis*) (Davis et al., 2019; Gunn-Moore et al., 2017; Stylianaki et al., 2019).

Among toothed whales, the beaked whales (BW; family Ziphiidae) are particularly cryptic and comprise more than 22 different species (Madsen et al., 2014). BW routinely dive deeper than 1000 m for about 1 h in order to hunt deep-water squids and fishes (Madsen et al., 2014). In fact, a Cuvier's beaked whale (*Ziphius cavirostris*) broke the diving record with a 3000 m-depth dive that lasted for 2 h (Madsen et al., 2014); this is by far the deepest dive recorded for any air-breathing endotherm animal.

Diving mammals are regularly exposed to hypoxic conditions during breath-hold diving and a large body of literature has addressed how they maintain their body functions (Cozzi et al., 2017; Fahlman et al., 2006; Ponganis, 2015; Tian et al., 2016). These mammals rely on a large blood volume rich in hemoglobin and in skeletal muscles with high concentrations of myoglobin, resulting in an enhanced capacity for tissue oxygen storage (Ponganis, 2015). Neural globin proteins, neuroglobin and cytoglobin, facilitate the movement of oxygen from blood to neural tissues (Williams et al., 2008). Neuroglobin is involved in enhancing primary hypoxic tolerance in the diving brain (Schneuer et al., 2012), scavenging reactive oxygen and nitrogen groups and so defending against cellular damage (Fago et al., 2004). Hence, during evolution, cetaceans should then have increased their tolerance to hypoxia and decreased metabolism during dives. Nevertheless, neuroglobin levels are not higher in diving mammals than in non-diving mammals (Larson et al., 2014).

In the last years, BW received public attention after a series of mass strandings in the Canary Islands, caused by mid-frequency naval sonars (Fernández et al., 2013; Fernández et al., 2017, 2012). Naval mid-frequency sonar have long been implicated in mass strandings and decompression-like sickness (DCS) and ceased when the Spanish government imposed a moratorium on naval exercises and the European Parliament issued a non-binding resolution in 2004 to stop the use of high-intensity sonar (Fernández et al., 2013). A couple of years later, the following statement was published: “Not even one euro nor one single dollar would have been bet by any Scientist, I guess, regarding the possibility that beaked whales, deep diving cetaceans belonging to the family Ziphiidae, could develop ‘Gas and Fat Embolic Syndrome’ (GFES) a condition mimicking ‘Decompression Sickness’ (DCS) in human divers. (…) Therefore, GFES-affected beaked whales could (should?) be regarded as ‘animal models’ for the study of DCS, their human disease ‘counterpart’.” (Di Guardo, 2018b).

## RESULTS

Herein, we present immunohistochemical evidence of CNS-positive labeling for Aβ and NFT in six out of nine (67%) animals, representing three odontocete species (Table 1).

**Table 1.**
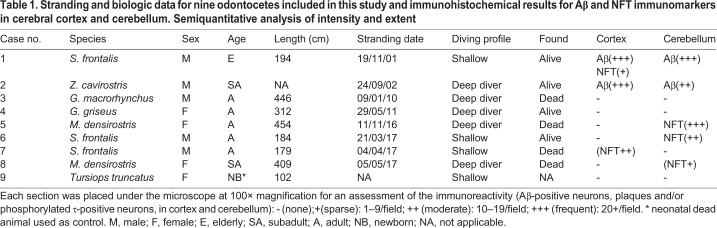
**Stranding and biologic data for nine odontocetes included in this study and immunohistochemical results for Aβ and NFT immunomarkers in cerebral cortex and cerebellum. Semiquantitative analysis of intensity and extent**

**Fig. 1.**
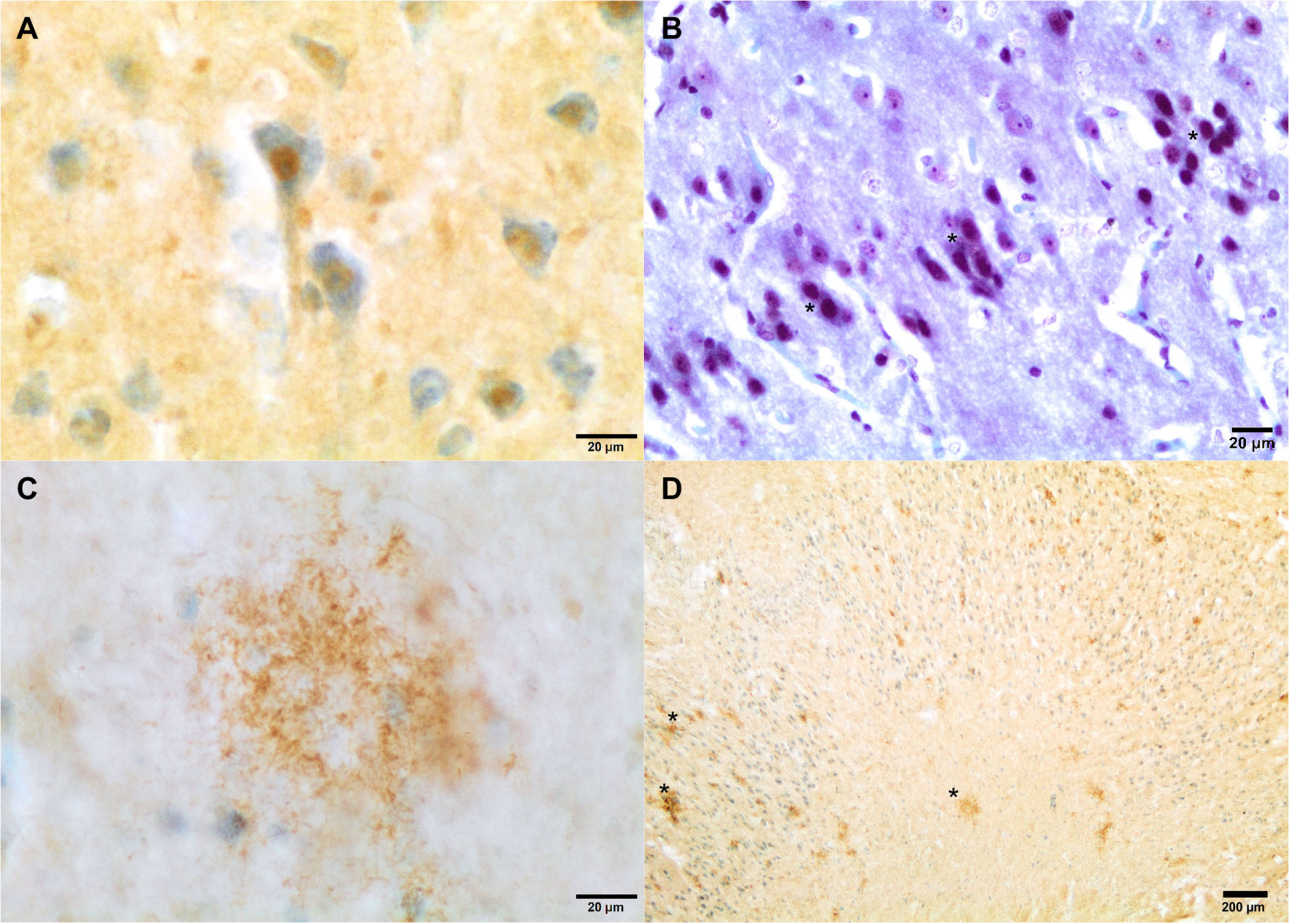
**Case 1 (*S.frontalis*).** Strong and diffuse nuclear staining to Aβ in the cortical neurons. Aβ free-floating immunolabeling; DAB not counterstained with thionine (A) Bennhold's Congo Red stain (B, asterisks). Scale bars: 20 µm. Aβ-positive aggregate in the frontopolar cortex (C). There are multiple irregular, slightly well-demarcated dense Aβ-positive extracellular/neuroparenchymal aggregates (D, asterisks) in the gray matter and subcortical white matter. Aβ free-floating immunolabeling; DAB counterstained with thionine.

**Fig. 2.**
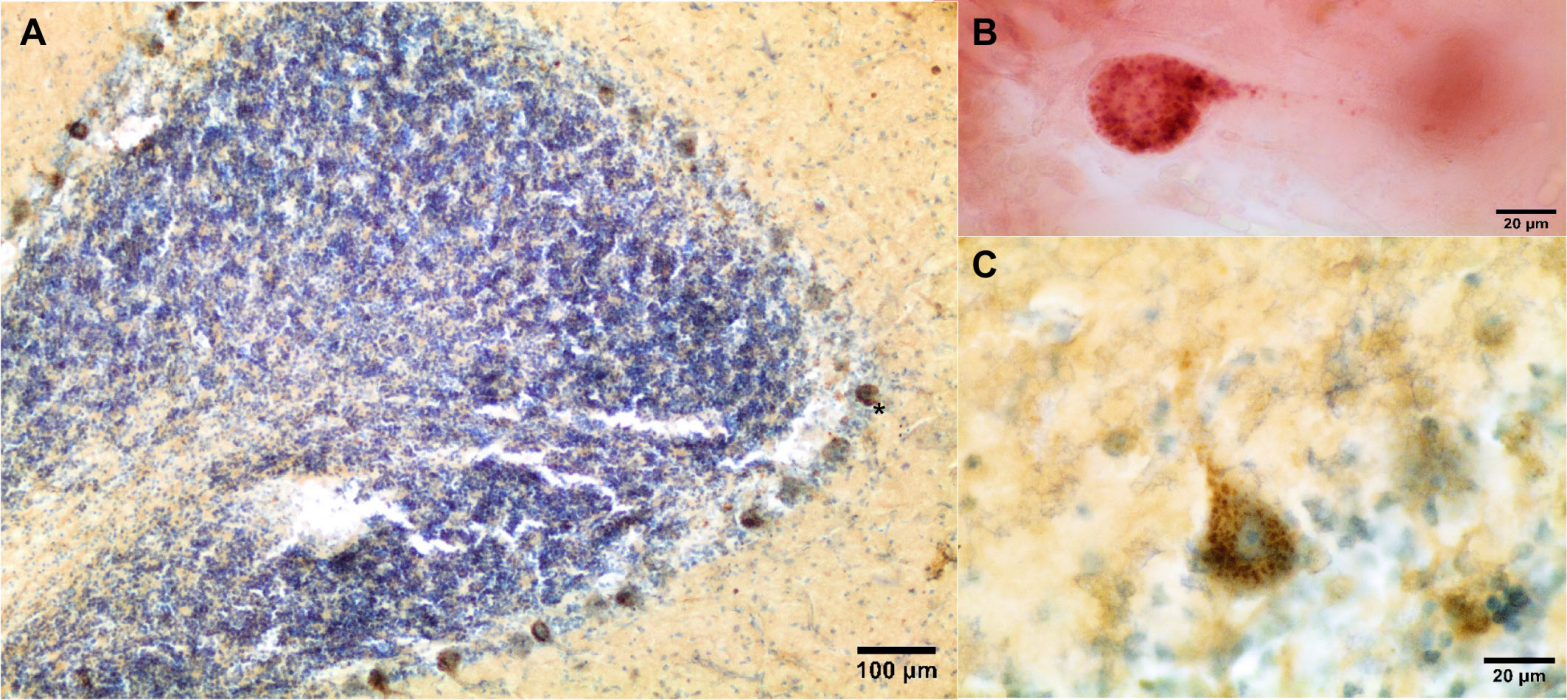
**Case 5 (*M.densirostris*).** NFT-granulovacuolar labeling in Purkinje neurons (A–C). Purkinje cells have focal to diffuse granular cytoplasmic NFT-positive labeling (A–C; A, asterisk). Free-floating immunolabeling; DAB counterstained with thionine (A,C). AEC not counterstained (B).

Case 1 (*Stenella frontalis*, elderly, Fig. 1) presented Aβ plaques in the frontopolar cortex. Also, there was strong and diffuse nuclear Aβ labeling throughout the five neuronal layers of the frontopolar cortex involving the three types of neurons: pyramidal, granular, and von Economo neurons, a type of large spindle-shaped neurons in layer V. Intranuclear staining was also detected in Purkinje neurons of the anterior and posterior cerebellum. Vascular Aβ deposition in the walls of cerebral vessels was also patent. Finally, the frontopolar cortex had scattered cortical neurons with diffuse cytoplasmic NFT labeling. Case 2 (*Z.* *cavirostris*, subadult) had moderate nuclear staining to Aβ in the neurons of the frontal neocortex and in the cerebellar Purkinje neurons. Case 5 (*Mesoplodon densirostris*, adult, Fig. 2) had widespread granulovacuolar cytoplasmic labeling to NFT in Purkinje neurons. This animal also presented cerebral nasitremiasis confined to the forebrain (unpublished data). Case 6 (*S.* *frontalis*, adult) had mild, multifocal granular to vacuolar, cytoplasmic labeling to NFT in scattered Purkinje neurons. Case 7 (*S.* *frontalis*, adult) had dispersed cortical neurons with diffuse cytoplasmic NFT labeling. Case 8 (*M.* *densirostris*, subadult) had scattered neurons with granular to vacuolar cytoplasmic labeling for NFT in Purkinje neurons.

Aβ immunopositivity involved cerebral and cerebellar neurons and Aβ plaques, and NFT immunopositivity was confined to cerebellar Purkinje neurons with granulovacuolar degeneration as well as in the cytoplasm of scattered frontopolar neurons as diffuse staining. These findings were more extensive in BW: one Cuvier's beaked whale (*Z. cavirostris*) and two Blainville's beaked whales (*M. densirostris*). However, they were also found in one elderly and two adult shallow-diver species, namely Atlantic spotted dolphins (*S. frontalis*). Two of three BW positive for Aβ and NFT were subadults and the other was an adult.

## DISCUSSION

### Aβ and hypoxia

**Fig. 3.**
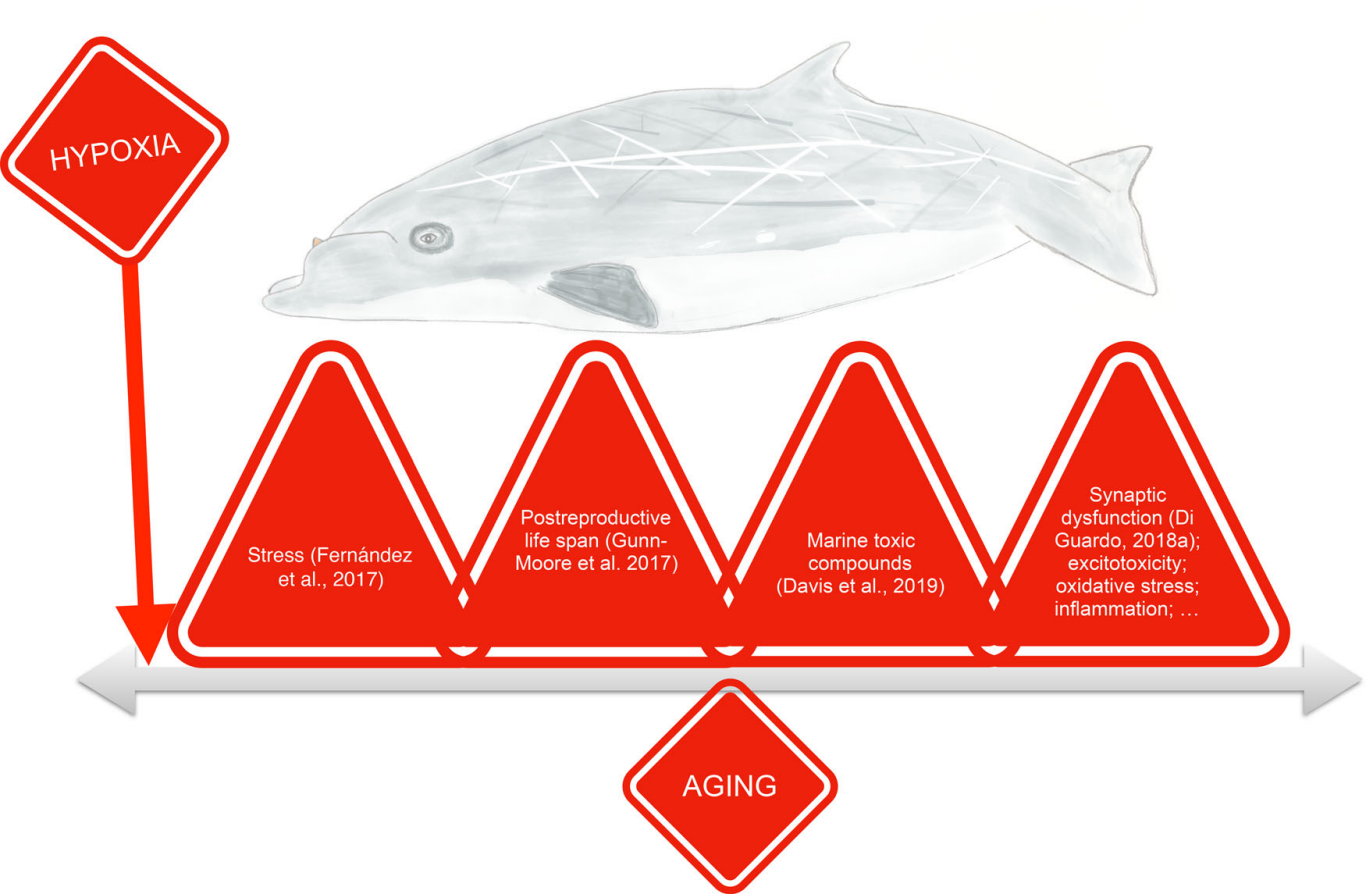
**Neurodegenerative diseases in diving marine mammals (here represented a beaked whale) may result from the interactive effects of multiple risk factors among which hypoxia could be one of the most important.**

Based on these results, we present a novel hypothesis that considers hypoxia as one of the most important risk factors that could contribute to NDD in cetaceans, with special attention to BW, alongside to other proposed risk factors/causes proposed by other authors (Davis et al., 2019; Di Guardo, 2018a, 2019; Fernández et al., 2017; Gunn-Moore et al., 2017) (Fig. 3). Hypoxia has been pointed out as a risk factor that may accelerate AD pathogenesis by altering Amyloid beta Precursor Protein (APP) processing. Moreover, repeated hypoxia increases amyloid generation and neuritic plaques formation and also activates macroautophagy (Li et al., 2009). Also, a gradual decline of oxygen and glucose supply to the brain during aging or hypoxic conditions has been demonstrated as a contributing factor to hypometabolism (Ashok et al., 2017). In AD patients, the brain regions with hypometabolism can trigger overexpression of APP and decrease the clearance of Aβ. Aβ and hypoxia can evoke inflammation, oxidative stress and eventual neuronal cell death (Ashok et al., 2017).

### Intranuclear neuronal Aβ-labeling: what does it mean?

Intranuclear neuronal Aβ-labeling is barely described in the literature. The cell nucleus has been pointed out as the site of amyloid-like protein fibrillation in different disorders recognized as widespread aggregation of proteins with instable homopolymeric amino acid repeats, ubiquitin, and other proteinaceous components (von Mikecz, 2014). In fact, Aβ neuronal inclusions have prion-like properties (Olsson et al., 2018) and are able to enter the nucleus through nuclear pores complex across the nuclear envelope, but only Aβ 42 plays a role in gene transcription (Barucker et al., 2014). It has been suggested that Aβ is deposited in the vicinity of DNA in the nuclear region of AD cells (Hegde et al., 2004). Intranuclear neuronal Aβ-labeling was observed in two cases in this study: an elderly *S. frontalis* and a subadult *Z. cavirostris*. The later was one of the 14 BW stranded in close temporal and geographic association with an international naval exercise (Neo-Tapon) held on September 24th, 2002 (Arbelo et al., 2013; Fernández et al., 2005). In this BW, we propose intranuclear neuronal Aβ expression having a neuroprotective role to hypoxia. While it was initially thought that nuclear amyloid is responsible for neural cell death, time-resolved experiments that correlate nuclear amyloid and neurodegeneration on the single cell level also suggest a cell protective role (von Mikecz, 2014). Even though the mechanism is still unknown and there is no confirmation to date on Aβ binding to DNA, the nuclear localization of Aβ might play a role in bringing about changes in DNA topology, modulating both helicity and superhelicity in super coiled DNA (Hegde et al., 2004). Although the role of nuclear amyloid is still unknown, the Aβ intranuclear expression provides new insights regarding cerebral safeguards for hypoxic-ischemic brain injury from accidents or disease.

### Granulovacuolar degeneration for selectively vulnerable neurons

Neuronal granulovacuolar degeneration (GVD) is one of the histopathological hallmarks of AD and is defined as electron-dense granules (0.5 to 1.5 mm in diameter) within double membrane-bound cytoplasmic autophagic vacuoles, mainly in the hippocampal pyramidal neurons (Braak and Braak, 1991). GVD is not pathognomonic of AD and can be observed in other NDD and in brains of non-demented elderly people (Köhler, 2016). The granules are composed of several components including neurofilament proteins, ubiquitin, phosphorylated τ, and other microtubule-associated proteins (Yamazaki et al., 2011). To the best of the authors' knowledge, GVD has not been observed in the very few studies performed in aged rats, wolverines and dogs (Is et al., 2007; Roertgen et al., 1996; Yu et al., 2011). Even though, in the gray mouse lemur, τ proteins were observed as clumps of thick granules located close to the membrane of the neuronal perikarya and the dendrites (Bons et al., 2006). GVD was observed in the Purkinje neurons of the anterior and posterior cerebellum of two BW and one *S.* *frontalis*. In the absence of ultrastructural studies, we can say that several antibodies to τ inconsistently label GVD indicating that the pattern of GVD immunoreactivity shows a phosphorylation state (hyperphosphorylation) of the granules that is similar to that of NFT (Köhler, 2016). Their role is not yet resolved and may be interpreted as either a cellular defense mechanism or an indicator of impaired cellular functioning (Köhler, 2016). Regardless of whether GVD is a cellular defense mechanism or not, this finding has never been documented in any marine mammal species (Thal et al., 2011). Future studies should address potential key roles of the hippocampal formation – so tiny in cetaceans – in NDD in marine mammals (Silvagni et al., 2005). In fact, certain structures of the hippocampus are vulnerable as are the Purkinje neurons of the cerebellum (Brierley, 1977). This would provide further support to ‘selectively vulnerable’ regions of the brain under hypoxic conditions.

### Concluding remarks

We strive to provide histopathological and immunohistochemical commonalities with human NDD, including AD, and surmise some factors, particularly hypoxia, may play a role in their neuropathogenesis. This study presents the first description of Aβ and NFT in the brain of BW, adding also to the few descriptions of GVD in the brain of non-experimental animals, being specifically the first report of GVD in the cerebellum. Our findings could be linked to hypoxic phenomena, as they were more extensive in the brains of deep diver cetacean species, specifically BW, and not only in elderly individuals. Despite their adaptations, diving mammals could be vulnerable to sustained and repetitive CNS hypoxia. Future studies should also address potential neuroprotective adaptations in these species. Drawing a specific fingerprint-like pattern of the behavior of neuronal and non-neuronal components of the brain (Fernández et al., 2017) could help to better understand the pathogenesis of some NDD.

## MATERIALS AND METHODS

### Animals, brain processing methodology and histopathological analysis

One of the main challenges of working with cetaceans' brains is to establish a valid methodology for an optimal manipulation and fixation of such specimens, for neuroanatomical and neuropathological studies. Such difficulties are related to (1) their brain size, (2) the logistic difficulties and laboratorial complexity to achieve a large sample size from certain elusive species, such as the BW – here the Cuvier's beaked whale and the Blainville's beaked whale, and (3) the difficulties to obtain ‘extremely fresh’ brain samples from stranded individuals.

The brain including cerebrum, cerebellum, brainstem and spinal cord of eight animals representing six different odontocete species that stranded and died along the coastline of the Canary Islands (28°17′29″N, 16°37′44″W; Spain), were included in this study. Five deep-diver animals were included in the study: one Cuvier's beaked whale, two Blainville's beaked whales, one short-finned pilot whale (*Globicephala macrorhynchus*), one Risso's dolphin (*Grampus griseu*s). Additionally, four shallow divers were also added: three Atlantic spotted dolphins (*S.* *frontalis*), and finally one captive neonatal bottlenose dolphin, which died of natural causes, was examined as control (Table 1).

The required permission for the management of stranded cetaceans was issued by the environmental department of the Canary Islands' Government and the Spanish Ministry of Environment. None of these experiments were performed on live animals, nor were these animals euthanized for the purposes of this study.

The animals were of different age categories: elderly (*n*=1), adult (*n*=5), subadult (*n*=2), newborn (*n*=1), based on total body length, gross and microscopic gonadal appearance (Geraci and Lounsbury, 2005), and systemic gross and microscopic features, e.g. pronounced tooth wear, neuronal lipofuscinosis, greater amount of neuromelanin in the locus coeruleus (Sacchini et al., 2018) or substantia nigra, intraneuronal polyglucosan bodies, leptomeningeal fibrosis and choroid plexus hyalinosis.

Brains were removed from the neurocranium carefully and promptly immersion-fixed at necropsy in 4% formaldehyde solution in phosphate-buffered saline (PBS; pH 7.4) and processed for neuroanatomical and neuropathological analysis as described by Sacchini et al. (2018). After rinsing in PBS, samples were cryoprotected in 30% sucrose solution in PBS (pH 7.4) at 4°C, to avoid freezing artifacts. The samples were immersed in a mixture of PBS-sucrose and Optimum Cutting Temperature formulation (OCT) (1:1) overnight. The day after, the samples were included in a mold using the same mixture, were rapidly frozen and 50–60 μm-thick serial sections were obtained employing a cryostat (Leica CM1950, Nussloch, Germany). Sections were stored in PBS (pH 7.4) solution with sodium azide (0.01%).

Additionally, for microscopic analysis, formalin-fixed, paraffin-embedded (FFPE) tissue sections were processed as routine, cut at 5 μm-thick and stained with Hematoxylin and Eosin.

### Immunoperoxidase staining

The immunoperoxidase staining procedure was carried out on free-floating sections as described in Sacchini et al. (2018). Commercially available, human-oriented primary antibodies (pAbs) used were a monoclonal anti-Aβ (1:100, clone H31L21, Invitrogen, Carlsbad, CA, USA), and a polyclonal anti-NFT (1:100, AHB0161, Invitrogen) incubated 48 h at 4°C. To block non-specific binding, sections were incubated in a solution containing normal goat serum (S-1000, Vector Laboratories, Burlingame, CA, USA), and 0.5% Triton X-100 (Merck, Darmstadt, Germany). The sections were then incubated for 45 min with a biotinylated goat anti-rabbit antibody (BA-1000, Vector Laboratories) diluted 1:200 in a solution containing 1% normal goat serum in PBS. No block for endogenous biotin was used. The immunoreactions were visualized either by 3,3′-diaminobenzidine (DAB) peroxidase kit (SK-4100, Vector Laboratories, city, country) followed by counterstaining with thionine, or by 3-amino-9-ethylcarbazole (AEC) peroxidase kit (Vector Laboratories, SK-4200) without counterstaining.

### Bennhold's Congo Red stain

Congo Red is the most popular dye used as a probe for diagnosing amyloidosis also in AD brains. The Bennhold's Congo Red stain is commonly used for the detection of amyloid on FFPE and frozen tissue sections (Gordon et al., 2006). The amyloid deposits are stained red and the nuclei are stained blue. FFPE samples were deparaffinized with xylene and rehydrated in graded ethanol. Sections were stained with Congo Red solution for 1 h, rinsed in distilled water, differentiated quickly in alkaline alcohol solution, and counterstained with Mayer's Hematoxylin. The positive control was a kidney from a dog with severe renal amyloidosis. The specific patterns of intraneuronal Aβ and granular (and in a few cases, diffuse) NFT labeling would exclude the possibility of cross-reactivity of shared epitopes. Additionally, the specificity of the Aβ uptake was attested to by comparison with Congo Red staining; there was complete correlation between the Congo Red staining visualized in the neuronal nuclei and the reference compound Aβ. Moreover, the Congo Red staining validated the immunopositivity not only in the elderly plaques but also in the nuclei of the neurons and confirmed the Aβ uptake.

### Semiquantitative scoring of Aβ and phosphorylated τ

Semiquantitative analysis of intensity and extent was performed manually on a light microscope (OLYMPUS BX41). The number of containing Aβ-positive neurons, plaques and/or phosphorylated τ-positive neurons in cortex and cerebellum, were counted with a 100× magnification in at least two fields of view from all positive animals. The semiquantitative scoring system was originally developed for neuritic plaques (CERAD score) and uses the categories absent when the score is none (−) or sparse (+) and present when the score is moderate (++) or frequent (+++) (Hyman et al., 2012). The immunoreactivity was rated as the following grading criteria were applied: sparse=1–9/field positive neurons or plaques, moderate=10–19/field positive neurons or plaques, frequent=more than 20/field positive neurons or plaques. The only animal with frequent Aβ plaques also contained both moderate vascular Aβ deposits as well as frequent intranuclear Aβ immunoreactivity in the cerebral cortex and cerebellum.
